# Τhe Effect of Opioid Administration on Cytologic and Histopathologic Diagnosis of Canine Cutaneous Mast Cell Tumors Treated by Surgical Excision

**DOI:** 10.3390/vetsci9050202

**Published:** 2022-04-22

**Authors:** Christina Marouda, Tilemahos Anagnostou, Ioannis Savvas, Lysimachos G. Papazoglou, Dimitra Psalla

**Affiliations:** 1 Laboratory of Pathology, School of Veterinary Medicine, Faculty of Health Sciences, Aristotle University of Thessaloniki, 54124 Thessaloniki, Greece; cmarouda@vet.auth.gr; 2 Unit of Anaesthesiology and Intensive Care, Companion Animal Clinic, School of Veterinary Medicine, Faculty of Health Sciences, Aristotle University of Thessaloniki, 54627 Thessaloniki, Greece; tanagnos@vet.auth.gr (T.A.); isavas@vet.auth.gr (I.S.); 3 Unit of Obstetrics and Surgery, Companion Animal Clinic, School of Veterinary Medicine, Faculty of Health Sciences, Aristotle University of Thessaloniki, 54627 Thessaloniki, Greece; makdvm@vet.auth.gr

**Keywords:** cutaneous mast cell tumors, opioids, histopathologic grading, cytologic grading, dog

## Abstract

Mast cell tumor (MCT) is a frequent cutaneous tumor in dogs, with a variable biological behavior. Studies correlate cytologic and histopathologic features of MCTs with their biological behavior, prognosis, and response to treatment. The use of preoperative opioids is common in canine patients undergoing surgical removal of these tumors. Certain opioids can induce or downregulate mast cell degranulation and influence cancer progression. The aim of the present study was to investigate whether the administration of morphine or butorphanol during surgical excision of canine cutaneous MCTs affects their cytologic and histopathologic appearance, thus influencing cytologic and histopathologic grading. This was a prospective, blinded, randomized, cohort clinical study. Forty-five dogs with cutaneous MCTs were randomly allocated into three groups according to preanaesthetic medication: dexmedetomidine combined with morphine (group M) or butorphanol (group B) or normal saline (group C). Cytologic specimens and histopathologic samples were obtained both prior to and after surgery. Samples were graded according to Kiupel’s and Patnaik’s systems, examined immunohistochemically for Ki-67 protein (Ki-67) and c-kit proto-oncogene product (KIT) expression, and histochemically for argyrophilic nucleolar organizing regions (AgNORs). Based on both Kiupel’s and Patnaik’s systems, no statistically significant differences were noted concerning the number of cases with grading discrepancies in grades allocated prior to versus after surgery among the groups. The same applied for cytological grading and immunohistochemical and histochemical evaluation. It seems that administration of morphine or butorphanol as part of the preanesthetic medication for surgical removal of canine cutaneous mast cell tumors does not influence histopathologic and cytologic grading of MCTs.

## 1. Introduction

Mast cell tumors (MCTs) are the most common and frequently diagnosed malignant skin tumors in dogs, accounting for 7 to 21% of all skin neoplasms [1,2,3]. The clinical presentation, gross appearance, and biological behavior of canine cutaneous MCTs varies widely [4]. They may range from well-circumscribed, easy-to-excise, solitary nodules, to multiple tumors that appear grossly aggressive. Highly malignant systemic disease has rarely been reported in dogs [5]. Fine needle aspiration (FNA) cytology can provide a straight-forward diagnosis of these tumors in almost all cases. Their distinctive intracytoplasmic granules can be observed in smears, from even poorly differentiated neoplasms [6].

However, histopathologic grading has been considered a cornerstone in determining the prognosis and appropriate therapeutic interventions for canine MCTs. The most widely utilized histopathologic grading schemes are the 3-tier scheme introduced by Patnaik et al. in 1984 [7] and the 2-tier scheme developed by Kiupel et al. in 2011 [1]. Specifically, in Patnaik’s grading scheme cutaneous MCTs are classified as well-differentiated (grade I), intermediate grade (grade II) and undifferentiated (anaplastic, grade III) neoplasms based on defined morphologic criteria with the degree of granulation being one of the most characteristic [7,8]. Histopathologic grading based on Patnaik’s system has been significantly correlated with overall survival [7,8,9,10,11]. Kiupel’s grading system classifies MCTs as either low or highly malignant based only on cell morphology and mitotic index. Significant correlations between histopathologic grading based on Kiupel’s grading system and survival time, possibility of recurrence and metastases have been established [1,10]. The degree of granulation of the MCTs is a key criterion for a cytologic classification proposed by Camus et al. [12] based on the 2-tier Kiupel’s histopathologic system [1]. Clinical data have shown that cytologic grading helps in the initial assessment and clinical approach, but there are reported limitations [13].

Apart from histopathologic grading, other indexes of cellular proliferation have been used to predict an even more accurate prognosis. These methods include histochemical staining for argyrophilic nucleolar organizing region (AgNOR), immunohistochemistry for Ki-67 protein (Ki-67) and routinely performed mitotic count (MI) [4,10,14,15,16,17]. The calculated product of Ki-67 and AgNOR scorings (Ki-67 × AgNOR) has been proposed to better reflect the total cellular proliferation within a tumor [3,14]. Another prognostic tool widely used is evaluation of c-kit proto-oncogene product/ tyrosine kinase receptor protein (KIT) localization through immunohistochemistry, in which deviated expression is correlated with decreased overall survival time, and increased incidence of local recurrence in canine cutaneous MCTs [18,19]. Therapeutic options for most cutaneous mast cell tumors include wide surgical excision, and/or adjuvant therapy including chemotherapy, tyrosine kinase inhibitor (TKI) therapy or radiation [20].

Opioids are commonly used as part of the preanesthetic medication for canine patients undergoing surgical removal of these tumors [21,22]. However, μ-opioid receptor agonists (e.g., morphine) may induce mast cell degranulation and cancer progression. These opioid actions have been studied in transgenic mice and in humans; however, there is no relevant study in dogs with MCTs [23,24]. On the other hand, κ-opioid receptor agonists/μ-opioid receptor antagonists (e.g., butorphanol) downregulate cutaneous mast cell activation and have an inhibitory role on tumor angiogenesis and tumor growth [25,26]. The degree of granulation of mast cells from MCTs plays a pivotal role in histopathologic and cytologic grading. Thus, it is likely that any factor that induces mast cell degranulation, also affects the histopathologic appearance of neoplastic cells and grading of such tumors. 

The aim of the present study was to investigate whether administration of certain opioids before surgical excision of canine cutaneous MCTs affects their cytologic and histopathologic features and influences cytologic and histopathologic grading and prognosis. In addition, we examined whether the sampling method used (incisional biopsy or excisional tissue biopsy) affects the histopathologic classification. The study’s hypothesis was that administration of morphine before surgical excision worsens cytologic and histopathologic grading compared to the grading obtained before administration, whereas administration of butorphanol before surgical excision does not alter the cytologic and histopathologic grading compared to the grading obtained before administration.

## 2. Materials and Methods

### 2.1. Animals

The study described here was conducted strictly in accordance with Νational and Εuropean animal welfare guidelines and was approved by the Institution’s Ethical Committee (567/13-3-2018). This was a prospective, blinded, randomized, cohort clinical study. It included dogs (and biopsy specimens and FNA samples obtained from these dogs) with cutaneous MCTs presented to the Companion Animal Clinic of Veterinary School, Aristotle University of Thessaloniki, Greece, during the period April 2017 to September 2020 for diagnosis and treatment of their cutaneous disease. Exclusion criteria included subcutaneous MCTs, administration of steroids, chemotherapy or antihistamines prior to surgery, and American Society of Anesthesiologists (ASA) physical status above II. Informed signed consent was obtained from all owners for participation of their animal in the study.

As part of the diagnostic approach, incisional skin biopsy specimens and FNA specimens from the lesions were obtained from all animals (histopathologic sample H1, cytologic sample C1). After completion of surgery (wide surgical excision with 2 cm margins of healthy tissue), the whole excised mass and FNA specimens were submitted for histopathologic and cytologic examination (histopathological sample H2, cytological sample C2). In cases with multiple MCTs, samples H1 and H2 and samples C1 and C2 were obtained from the same mass. Data recorded included breed, sex, age, tumor location as well as follow-up information (tumor recurrence or metastasis, adjuvant chemotherapy, survival, cause of death). 

### 2.2. Allocation into Groups and Anesthetic Management 

Dogs were randomly allocated into three groups according to preanesthetic medication: 0.15 mg/kg morphine (Morphine sulfate, Famar SA, Athens, Greece) combined with 150 μg/m^2^ dexmedetomidine (Dexmedetomidine hydrochloride, Dexdomitor, Zoetis, Kalamazoo, MI, USA) intramuscularly (IM) (group M) or 0.15 mg/kg butorphanol (Dolorex inj, MSD Animal Health, Intervet Hellas, Athens, Greece) together with 150 μg/m^2^ dexmedetomidine IM (group B), or 0.015 mL/kg normal saline (Sodium Chloride 0.9% Intravenous Infusion, Vioser, Trikala, Greece) together with 225 μg/m^2^ dexmedetomidine IM (group C), by means of a random numbers table. All dogs of all groups were subjected to complete blood count, serum biochemistry examinations, and a thorough preanesthetic clinical examination. Dogs were fasted for approximately 14 h after their last meal and had free access to water until 2 h before preanesthetic medication. 

On the day of surgery, the combinations of drugs according to group allocation were administered as preanesthetic medication. Lactated Ringer’s solution (L-R, Lactated Ringer’s Injection; Vioser, Trikala, Greece) was infused IV at a rate of 5 mL/kg/h^−^ after establishment of intravenous access until the end of surgery. Cefazolin (Vifazolin, Vianex, Athens, Greece) 20 mg/kg IV was administered at anesthetic induction. Propofol (Propofol MCT/LCT Fresenius, Fresenius Kabi Hellas, Athens, Greece) was used to induce anesthesia at a dose of 1 mg/kg IV, with additional doses given if needed to facilitate unhindered tracheal intubation with a cuffed endotracheal tube of appropriate size. Isoflurane (Forrane, Baxter Healthcare Ltd., Norfolk, UK) in 100% oxygen was used for maintenance of anesthesia administrated via an appropriate breathing system connected to an anesthetic machine. Depth of anesthesia was adjusted appropriately intra-operatively to match the intensity of surgical stimulation. The same surgeons performed surgery in all cases. Arterial blood pressure measurement (indirectly, oscillometry), electrocardiography (lead II), pulse oximetry (PC Scout; SpaceLabs Medical Inc., Redmond, WA, USA), side-stream capnography and measurement of inhaled and exhaled oxygen and isoflurane concentrations (Capnomac Ultima, Datex-Engstrom, Helsinki, Finland) were used for patient monitoring. Meloxicam (Metacam inj, Boehringer Ingelheim, Ingelheim am Rhein, Germany) 0.1 mg/kg IV was administered postoperatively. In case signs attributed to massive mast cell degranulation like persistent hypotension and/or local hemorrhage and/or diffuse erythema were noted, prednisolone (Prezolon inj, Takeda, Athens, Greece) 0.5 mg/kg was administered IV and meloxicam was omitted. 

### 2.3. Sampling

Histologic samples taken during the initial diagnostic approach (sample H1, before surgery) were obtained using a 6 mm biopsy punch in 8–10 mm depth (Biopsy punch; Kruuse, Langeskov, Denmark) under local anesthesia with lidocaine (subcutaneous injection into healthy tissue surrounding the neoplastic nodule). Initial biopsy tissues (samples H1) and the whole surgically excised tumors (samples H2) were fixed in 10% neutral buffered formalin and submitted for histopathologic and immunohistochemic evaluation. All cytologic samples (samples C1 and C2) were fixed in methanol solution.

### 2.4. Cytologic Grading

Cytologic samples were stained with May Grünwald–Giemsa. Mast cell tumors were classified according to the modified Kiupel’s 2-tier grading system proposed by Camus et al. for cytologic specimens [12]. Specifically, poorly granulated specimens and/or specimens, in which the presence of at least two malignancy criteria (presence of mitotic figures, nuclear pleomorphism, binucleation or multinucleation, or marked anisokaryosis) were noted, were classified as high grade. Well-granulated specimens with absence of the abovementioned malignancy features were classified as low grade. Any disagreements in malignancy grade assignment for any one specific case between grading C1 and C2 were noted, and the numbers of cases with grade discrepancies, as well as the direction of grade change (deterioration or improvement) for each group, were recorded.

### 2.5. Histopathologic Examination and Grading

Slides were prepared with 4-μm thick sections of formalin-fixed paraffin-embedded specimens and stained routinely with hematoxylin and eosin and with metachromatic toluidine blue dye to better highlight mast cell granules. Histopathologic grading was performed based on both existing grading systems, Patnaik’s (grades I, II, III) and Kiupel’s (low and high malignancy) [1,7]. The histopathologic grading criteria used by the two systems are summarized in Table 1. The grade in all specimens was assigned by the same examiner who was blinded to the group allocation. A histopathologic grade was assigned to the initial punch biopsy (samples H1 obtained before anesthesia and surgery) and to two different specimens originating from two different sites of the excised mass obtained after anesthesia and surgery, i.e., superficial area of the mass (samples H2a) and center of the mass (samples H2b). Any disagreements in grade assignment for any one specific case between grading H1 and H2a or between H1 and H2b or between H2a and H2b were noted. Τhe numbers of cases with grade discrepancies, as well as the direction of grade change (deterioration or improvement) for each group, were recorded. 

### 2.6. Immunohistochemistry for Ki-67 and KIT 

Immunohistochemical staining and evaluation for Ki-67 and KIT was conducted in the Laboratory of Pathology, School of Veterinary Medicine, Faculty of Health Sciences, Aristotle University of Thessaloniki, Greece, using techniques described in the literature [14]. Immunolabeling on positively charged slides for Ki-67 was performed using a monoclonal mouse anti-human antigen (Clone MIB-1, Dako, Glostrup, Denmark) at dilution 1:50 for one hour after epitope retrieval in EDTA microwave incubation (EnvisionFLEX, Target retrieval solution, high pH, Dako, Glostrup, Denmark) for 20–30 min at 500 watts (95–100 °C). Detection of primary antibody binding was done using the Ultra Vision Quanto Detection system HRP DAB (DAB Quanto chromogen, Epredia, Montréal, Quebec, Canada) and slides were counterstained with hematoxylin. For KIT labeling, a polyclonal rabbit anti-human antibody (CD117, Dako, Glostrup, Denmark) was used, in 1:400 dilution at room temperature for one hour, whereas heat-retrieval method, antibody detection and counterstaining were the same as mentioned above. As negative controls, samples from confirmed cases of canine cutaneous MCTs were included in each run, and they were treated identically to the other tissue sections except that buffer was used in place of primary antibody. Known sections of canine cutaneous MCTs were also included in each run as positive controls for KIT. The basal layer of the epidermis of the same samples served as an internal positive control for Ki-67.

### 2.7. Ki-67 Scoring and KIT Patterns

For Ki-67 scoring, cell counting in all samples from all groups was performed manually using a 1 cm^2^ 10 × 10 mm grid reticle adapted in a microscope at 400× magnification. Areas with the highest amount of immunohistochemically positive mast cells were selected from H1 and from H2 (with H2a and H2b areas considered together) samples, and the total numbers of positive-staining nuclei were calculated as previously described [27]. Positive nuclei were counted in five grid areas in each sample. Using the cutoff value of 23 that has been determined previously by Webster et. al. [14], and based on the estimated mean value of the samples of the present study, samples were classified as high- or low-malignancy samples. Any disagreements in malignancy assignment for any one specific case between H1 and H2 Ki-67 classification were noted, and the numbers of cases with malignancy assignment discrepancies, as well as the direction of malignancy assignment change (deterioration or improvement) for each group, were recorded.

For KIT expression classification, the area with the greatest KIT positivity was chosen at 100× magnification in H1 and H2 (with H2a and H2b areas considered together) samples. Cases were categorized into one of three KIT patterns, as described by Webster et al. [14]. Specifically, KIT pattern I is described as peri-membrane labeling, KIT pattern II is described as focal perinuclear or stippled cytoplasmic localization, and KIT pattern III is described as diffuse cytoplasmic localization in >10% neoplastic cells [14,18]. Any disagreements in malignancy assignment for any one specific case between H1 and H2 KIT classification were noted, and the numbers of cases with malignancy assignment discrepancies, as well as the direction of malignancy assignment change (deterioration or improvement) for each group, were recorded.

### 2.8. AgNOR Histochemical Staining and Evaluation

Positively charged slides were prepared with 4 μm thick sections of formalin-fixed paraffin-embedded specimens, and AgNOR staining was performed according to the silver staining method introduced by Ploton et al. [28]. AgNORs were counted in 100 randomly selected neoplastic mast cells as observed in 1000× magnification. AgNOR’s mean values from H1 and from H2 (with H2a and H2b areas considered together) samples were determined, and the product of Ki-67 × AgNOR was also calculated. Using the cutoff value of 54 that has been determined previously by Webster et al. [14] and based on the estimated mean value of the samples of the present study, samples were classified as high- or low-malignancy samples. Any disagreements in malignancy assignment for any one specific case between H1 and H2 Ki-67 × AgNOR classification were noted, and the numbers of cases with malignancy assignment discrepancies, as well as the direction of malignancy assignment change (deterioration or improvement) for each group, were recorded.

### 2.9. Statistical Analysis

Power analysis was performed, and a total sample size of 43 was calculated in order to achieve a 0.8 power of detecting a 50% change in grade assignment. Analysis of variance (ANOVA) was used to evaluate any differences among the groups regarding weight and age of the animals. The Kolmogorov–Smirnov test was used to evaluate normality. The Z-test for differences between population proportion was used to evaluate any differences between the proportions of the grading changes. The level of statistical significance was set at α ≤ 0.05. All statistical analyses were performed using the software package IBM SPSS Statistics, Version 27.

## 3. Results

### 3.1. Animals

Forty-five dogs were included in the study. Twenty-nine of the dogs were males and 16 females. There were five mixed-breed dogs and 40 purebred dogs (seven Boxers, six Labradors, five Golden Retrievers, five Pit Bulls, four French Bulldogs, three English Setters, three Maltese, two Brittany Spaniels, one American Staffordshire terrier, one English Bulldog, one Pincher, one Pug and one Yorkshire Terrier). The dogs’ age ranged from 2 to 15 years, with a median of eight years, and their bodyweight ranged from 4 to 39.7 kg, with a median of 25 kg. Concerning age and bodyweight, the three groups differed statistically non-significantly. Tumor locations included the head (nasal planum and upper lip) and neck, inguinal area, scrotum, interdigital area, perianal area, lateral thoracic wall, and sternum. A solitary mass was detected in 23 cases, whereas multiple MCTs were identified in the remaining 22 cases.

### 3.2. Cytologic Grading

Cytologic gradings for all samples (C1 and C2) are listed in Table 2. Only in a single case of group M, cytological characterization changed to high malignancy on C2 sample when compared to the low-malignancy characterization allocated to the respective C1 sample. No cases of disagreement in characterization between C2 and C1 were noted in groups B and C, and a statistically non-significant difference was found (*p* = 0.2335).

### 3.3. Histopathologic Grading

Histopathologic grades for Patnaik’s and Kiupel’s systems for samples H1, H2a and H2b are listed in Table 3. Based on Patnaik’s classification system, the histopathologic grade assigned in H2b samples when compared to the grade allocated to the respective H1 samples changed in six out of the 15 cases of group M, five out of the 12 cases of group B, and five out of the 18 cases of group C, with the differences being statistically non-significant (*p* ≥ 0.2934). In all cases, the histopathologic evaluation led to a more malignant characterization on H2b samples. Based on Kiupel’s classification system, H2b samples changed to high malignancy when compared to the characterization of low malignancy allocated to the respective H1 samples, in two out of the 15 cases of group M, one out of the 12 cases of group B, and one out of the 18 cases of group C, with the differences being statistically non-significant (*p* ≥ 0.3001). 

Based on both Kiupel’s and Patnaik’s systems, no disagreement in the histopathologic grade assigned was noted between samples H1 and H2a in any group.

**Figure 1:**
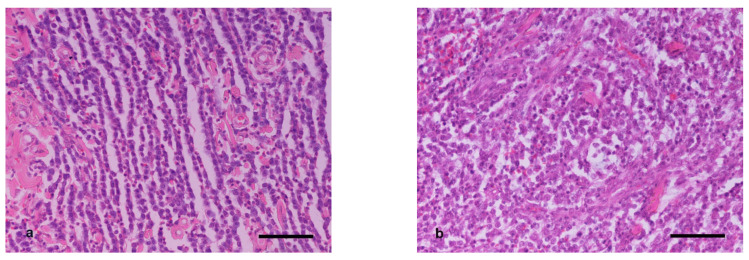
H&E staining (**a**) Low-grade canine MCT from a H2a sample (surface layers). Well-differentiated neoplastic mast cells with minimal pleomorphism and low mitotic index arranged in rows. (**b**) High-grade MCT from the H2b area (center of the excised mass) of the same case as in (**a**). Increased anisokaryosis and mitotic index of neoplastic mast cells. Scale bar: 100 μm.

Based on Patnaik’s classification system, the comparison of histopathologic grade allocated for samples H2a to grades for samples H2b showed that changes occurred in six out of the 15 cases of group M, five out of the 12 cases of group B, and five out of the 18 cases of group C, with the differences being statistically non-significant (*p* ≥ 0.2934). In all cases, the histopathologic evaluation led to a more malignant characterization on H2b samples (Figure 1). Based on Kiupel’s classification system, H2b samples changed to high malignancy when compared to the characterization of low malignancy allocated to the respective H2a samples in two out of the 15 cases of group M, one out of the 12 of group B, and one out of the 18 cases of group C, with the differences being not statistically significant (*p* ≥ 0.3001).

### 3.4. Kit Pattern

KIT pattern for all samples (H1 and H2) are listed in Table 4. Concerning the comparison of H2 pattern allocation to H1 pattern allocation, in group M, only one out of the 15 samples changed pattern (from I to II). Similarly, in group C, one out of the 18 samples changed pattern (from I to II). No change in KIT pattern was observed in group B. Statistical analysis of the results concerning the number of disagreements in KIT pattern characterization between samples H1 and H2 showed that there were statistically non-significant differences among the 3 groups (*p* ≥ 0.05). 

### 3.5. Ki-67 and AgNOR Counts

**Figure 2:**
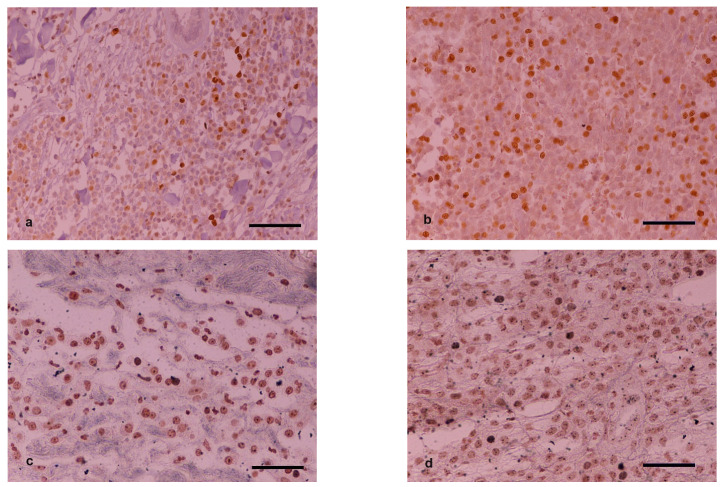
Ki-67 immunohistochemical staining, DAB chromogen, H&E counterstain (**a**,**b**). (**a**) MCT with low Ki-67 scoring (**b**) MCT with high Ki-67 scoring. Histochemical staining for argyrophilic nucleolar organizing region (AgNOR) (**c**,**d**). (**c**) MCT with low AgNOR scoring (**d**) MCT with high AgNOR scoring. Scale bar: 50 μm.

Characterization of samples based on average Ki-67 and AgNOR counts and their calculated product using predefined cutoff values are listed in Table 5. In group M, two out of the 15 cases changed Ki-67 counts on H2 scoring compared to H1 so as to overcome the cutoff value of 23 and thus change malignancy characterization. The corresponding Ki-67 × AgNOR counts for these two cases did not lead to a different characterization on H2 compared to H1 samples (remained on the same side of the 54-cutoff value). In group B, one out of the 12 cases changed Ki-67 and Ki-67 × AgNOR counts on H2 scoring compared to H1 so as to overcome the respective cutoff values and thus change malignancy characterization. In group C, one out of the 18 cases changed Ki-67 count on H2 scoring compared to H1 so as to overcome the cutoff value of 23 and thus change malignancy characterization. Furthermore, three out of the 18 cases changed Ki-67 × AgNOR counts on H2 scoring compared to H1 so as to overcome the cutoff value of 54 and thus change malignancy characterization (Figure 2). 

Statistical analysis of the results concerning the number of disagreements in malignancy characterization based on Ki-67 and Ki-67 × AgNOR counts between samples H1 and H2 showed that there were statistically non-significant differences between the groups (*p* ≥ 0.3001 and *p* ≥ 0.0659, respectively). 

## 4. Discussion

Morphine or butorphanol or other opioids are commonly administered as part of the preanesthetic medication before the surgical excision of canine cutaneous MCTs, mainly because they are highly effective drugs for managing perioperative pain in those patients [29,30]. To date, several in vitro and animal studies have documented the effect of certain opioids on cancer progression and mast cell degranulation [24,31,32,33]. However, not all opioids share the same pharmacokinetic and pharmacodynamic characteristics and, as a result, variable tumor-modulating effects may occur. Specifically, morphine as a pure μ-opioid receptor agonist augments tumor growth in established neoplasms [24]. Butorphanol, acting as a κ-opioid receptor agonist and μ-opioid antagonist, seems to play an inhibitory role to tumor growth [26]. Morphine, among other stimuli, can induce cutaneous mast cell degranulation mainly through Mas-related G-protein-coupled receptor signaling and, to a lesser extent, through immediate activation of μ-opioid receptors [33,34]. κ-Opioid receptor agonists decrease mast cell degranulation in humans and mice [25]. 

Based on these data, we hypothesized that morphine could worsen the histopathologic and cytologic grading due to possible alterations in granularity and tumor progression of canine mast cell tumors after its administration, whereas butorphanol would not have the same effect. In the present study, cases in which a change was observed in the histopathologic and/or cytologic grading before and after the administration of opioids occurred in all groups, including the control group, and the proportion of such changes differed statistically non-significantly among groups. Thus, the results illustrate that administration of morphine as part of the preanesthetic medication in dogs undergoing cutaneous MCT surgical removal does not seem to influence histopathologic and cytologic grading compared to butorphanol or saline administration. 

The use of immunohistochemistry confirmed that the effect of morphine does not worsen the final assessment, neither the proliferation rate nor the expression of the receptor tyrosine kinase KIT. The results of the present study confirm that Ki-67, AgNOR, and KIT localization are expressed independently to the preanesthetic medication. Their prognostic value has been suggested in multiple studies [3,14,15,35]. 

Comparison of the grading of pretreatment biopsies (H1) with the grading of the central areas of the excised masses (H2b) showed few discrepancies in the histopathologic grading when using both grading systems in all groups. It is worth noting that neoplastic mast cells in the H1 samples, obviously obtained from the periphery of the masses, were histopathologically more “well differentiated” compared to cell populations in the center of the excised mass (H2b samples). Comparison of histopathologic grading from the pretreatment biopsies (H1) with the histopathologic grading from the superficial part of the excised mass (H2a) revealed no differences in any group. The same “well-differentiated” phenotype was detected in such areas, leading to the conclusion that the discrepancy was due to the sampling site (periphery vs. center of the mass). 

Variability in phenotypic and functional properties among cancer cells within the same tumor was described [36] and should be considered when examining biopsy samples to evaluate tumor malignancy. Reliability in the histopathologic grading of MCTs pretreatment biopsies to gain an accurate prognosis and plan therapeutic approaches is crucial. Shaw et al. concluded in a recent study that pretreatment biopsies are adequate to classify these tumors into low and high grade [37]. Similarly, to their conclusion, the present study confirmed only limited misclassified cases. In these cases, underestimation was always the problem. It seems that the low degree of granulation in the center of the tumor, where a bigger fraction of undifferentiated cell population appears to reside, facilitates observation of their nuclear characteristics (karyomegaly, presence of bizarre nuclei or multinucleated cells) and leads to better evaluation of tumor malignancy.

Concerning grading based on tumor markers, the present study revealed even fewer discrepancies between pretreatment biopsies and surgically removed tumor masses, highlighting the value of immunohistochemistry in prognostication and treatment plans of canine MCTs.

The degree of cell granulation in cytologic smears is crucial for the classification of the tumor into low- or high-malignancy categories [12,13]. According to observations by Hergt et al., examination of samples obtained via FNA of a degranulated or heavily granulated mast cell tumor either over- or underdiagnose high-grade MCTs, respectively [13]. Morphine-induced mast cell degranulation was expected to potentially alter the cytological appearance of FNA specimens obtained after administration. However, cases in which a change was observed in the cytologic grading before and after the administration of opioids in the present study were extremely few (only one case) and the proportion of such changes differed statistically non-significantly among groups. Thus, no visible and measurable “degranulation results” were noted. This observation is in accordance with a recent study which reported that morphine did not induce histamine release in in vitro canine MCT cell lines and did not affect plasma histamine concentrations in vivo [38]. Alterations in the cytologic image could have been induced not only by the effect of morphine, but also in response to surgical manipulations [39]. Nevertheless, the results of the present study indicate that degranulation due to surgical manipulations did not crucially change cytologic diagnosis. 

Although perioperative administration of H1-blockers is a relatively common clinical practice to reduce the risk of local and systemic effects of histamine release [40], many veterinary anesthetists believe that this treatment is pointless due to lack of histamine release during tumor manipulation and lack of evidence of effectiveness of antihistamine drugs. Even though histamine plasma concentrations were not measured in the present study, no dog exhibited signs that could be potentially attributed to massive mast cell degranulation, including persistent hypotension and/or local hemorrhage and/or diffuse erythema. 

## 5. Conclusions

In conclusion, it seems that administration of morphine or butorphanol as part of the preanesthetic medication for surgical removal of canine cutaneous mast cell tumors does not affect histopathologic and cytologic grading of MCTs. 

## Figures and Tables

**Table 1:** (**a**) Histopathologic criteria for Patnaik’s grading scheme. (**b**) Histopathologic criteria for Kiupel’s grading scheme.

**(a)**			
**Patnaik Classification System**			
**Histologic Grade**	**I**	**II**	**III**
Depth Infiltration/extent of tissue involvement	well delinated, confined to the dermis	lower dermis, subcutis or deeper tissues	Poorly circumscribed, cells in sheets that replace subcutis and underlying tissues
Cellularity	moderate	moderate to highly cellular	highly cellular
Cellular Morphology	well differentiated with round nuclei and metacromatic granules	moderately pleomorphic with finely granular cytoplasm	poorly granulated round to polygonal highly pleomorphic cells
Nuclear Pleomorphism	round nuclei with no distinct nucleoli	moderate, round nuclei with a single nucleolus	bi- or multinucleated cells, round vesiculated nuclei with 1 or more nucleoli
Mitotic Index	no mitotic figures	small numbers of mitotic figures, 0–2 hpf	3–6 per hpf
Stromal reaction	minimal	thick or hyalinized collagenous stroma with areas of oedema and necrosis	ulceration, necrosis and oedema
**(b)**			
**Kiupel Classification System**			
High malignancy	karyomegaly in >10% of neoplastic cells or > 3 cells with bizarre nuclei in 10 hpf or >3 multinucleated cells or >7 mitoses/10 hpf		
Low malignancy	If none of the above criteria applies		

**Table 2:** Cytologic grading allocated in samples C1 and C2.

Group	FNA Sample	Cytological Grading	
		Low	High
M (n = 15)	C1	8	7
	C2	7	8
B (n = 12)	C1	7	5
	C2	7	5
C (n = 18)	C1	10	8
	C2	10	8

M: Morphine group, B: Butorphanol group, C: control group; C1: FNA specimens before opioid or saline administration, C2: FNA specimens after opioid or saline administration.

**Table 3:** Histopathologic grading for Patnaik’s and Kiupel’s systems allocated in samples H1, H2a and H2b.

Group	Sample	Patnaik Classification			Kiupel Classification	
		I	II	III	Low	High
M (n = 15)	H1	4	11	0	11	4
	H2a	4	11	0	11	4
	H2b	0	13	2	9	6
B (n = 12)	H1	5	7	0	8	4
	H2a	5	7	0	8	4
	H2b	2	8	2	7	5
C (n = 18)	H1	10	8	0	16	2
	H2a	10	8	0	16	2
	H2b	5	13	0	15	3

M: Morphine group, B: Butorphanol group, C: control group; H1: pretreatment biopsies, H2a: surface of excised masses, H2b: center of excised masses.

**Table 4:** Characterization based on KIT distribution allocated in samples H1 and H2.

Group	Sample	KIT Patterns		
		I	II	III
M (n = 15)	H1	4	5	6
	H2	3	6	6
B (n = 12)	H1	3	3	6
	H2	3	3	6
C (n = 18)	H1	9	4	5
	H2	8	5	5

M: Morphine group, B: Butorphanol group, C: control group; H1: Pretreatment biopsies, H2: excised tumors (H2a and H2b areas of the mass considered together).

**Table 5:** Classification based on Ki-67 and Ki-67 × AgNOR scorings allocated in H1 and H2 samples, (predefined cutoff values of 23 and 54, respectively).

Group	Sample	Ki-67		Ki-67 × AgNOR	
		<23	>23	<54	>54
M (n = 15)	H1	14	1	14	1
	H2	12	3	14	1
B (n = 12)	H1	12	0	12	0
	H2	11	1	11	1
C (n = 18)	H1	16	2	18	0
	H2	15	3	15	3

M: Morphine group, B: Butorphanol group, C: control group; H1: Pretreatment biopsies, H2: excised tumors (H2a and H2b areas of the mass considered together).

## Data Availability

All data generated or analysed during this study are included in this published article. The datasets used and/or analysed during the present study are available from the first author upon reasonable request.

## References

[1] Kiupel M., Webster J.D., Bailey K.L., Best S., DeLay J., Detrisac C.J., Fitzgerald S.D., Gamble D., Ginn P.E., Goldschmidt M.H. (2011). Proposal of a 2-Tier Histologic Grading System for Canine Cutaneous Mast Cell Tumors to More Accurately Predict Biological Behavior. Vet. Pathol..

[2] Berlato D., Bulman-Fleming J., Clifford C.A., Garrett L., Intile J., Jones P., Kamstock D.A., Liptak J.M., Pavuk A., Powell R. (2021). Value, Limitations, and Recommendations for Grading of Canine Cutaneous Mast Cell Tumors: A Consensus of the Oncology-Pathology Working Group. Vet. Pathol..

[3] Sledge D.G., Webster J., Kiupel M. (2016). Canine cutaneous mast cell tumors: A combined clinical and pathologic approach to diagnosis, prognosis, and treatment selection. Vet. J..

[4] Kiupel M., Camus M. (2019). Diagnosis and Prognosis of Canine Cutaneous Mast Cell Tumors. Vet. Clin. N. Am. Small Anim. Pract..

[5] Welle M.M., Bley C.R., Howard J., Rüfenacht S. (2008). Canine mast cell tumours: A review of the pathogenesis, clinical features, pathology and treatment. Vet. Dermatol..

[6] Sabattini S., Renzi A., Marconato L., Militerno G., Agnoli C., Barbiero L., Rigillo A., Capitani O., Tinto D., Bettini G. (2018). Comparison between May-Grünwald-Giemsa and rapid cytological stains in fine-needle aspirates of canine mast cell tumour: Diagnostic and prognostic implications. Vet. Comp. Oncol..

[7] Patnaik A.K., Ehler W.J., Mac Ewen E.G. (1984). Canine Cutaneous Mast Cell Tumor: Morphologic Grading and Survival Time in 83 Dogs. Vet. Pathol..

[8] Stefanello D., Buracco P., Sabattini S., Finotello R., Giudice C., Grieco V., Iussich S., Tursi M., Scase T., Di Palma S. (2015). Comparison of 2- and 3-category histologic grading systems for predicting the presence of metastasis at the time of initial evaluation in dogs with cutaneous mast cell tumors: 386 cases (2009–2014). JAVMA.

[9] Hume C.T., Kiupel M., Rigatti L., Shofer F.S., Skorupski K.A., Sorenmo K.U. (2011). Outcomes of dogs with grade 3 mast cell tumors: 43 cases (1997–2007). J. Am. Anim. Hosp. Assoc..

[10] Horta R.S., Lavalle G.E., Monteiro L.N., Souza M.C.C., Cassali G.D., Araújo R.B. (2018). Assessment of Canine Mast Cell Tumor Mortality Risk Based on Clinical, Histologic, Immunohistochemical, and Molecular Features. Vet. Pathol..

[11] Murphy S., Sparkes A.H., Smith K.C., Blunden A.S., Brearley M.J. (2004). Relationships between the histopathological grade of cutaneous mast cell tumours in dogs, their survival and the efficacy of surgical resection. Vet. Rec..

[12] Camus M.S., Priest H.L., Koehler J.W., Driskell E.A., Rakich P.M., Ilha M.R., Krimer P.M. (2016). Cytologic Criteria for Mast Cell Tumor Grading in Dogs With Evaluation of Clinical Outcome. Vet. Pathol..

[13] Hergt F., von Bomhard W., Kent M.S., Hirschberger J. (2016). Use of a 2-tier histologic grading system for canine cutaneous mast cell tumors on cytology specimens. Vet. Clin. Pathol..

[14] Webster J.D., Yuzbasiyan-Gurkan V., Miller R.A., Kaneene J.B., Kiupel M. (2007). Cellular proliferation in canine cutaneous mast cell tumors: Associations with c-KIT and its role in prognostication. Vet. Pathol..

[15] Scase T.J., Edwards D., Miller J., Henley W., Smith K., Blunden A., Murphy S. (2006). Canine mast cell tumors: Correlation of apoptosis and proliferation markers with prognosis. J. Vet. Intern. Med..

[16] Romansik E.M., Reilly C.M., Kass P.H., Moore P.F., London C.A. (2007). Mitotic index is predictive for survival for canine cutaneous mast cell tumors. Vet. Pathol..

[17] Thamm D.H., Mauldin E.A., Vail D.M. (1999). Prednisone and vinblastine chemotherapy for canine mast cell tumor--41 cases (1992–1997). J. Vet. Intern. Med..

[18] Kiupel M., Webster J.D., Kaneene J.B., Miller R., Yuzbasiyan-Gurkan V. (2004). The Use of KIT and Tryptase Expression Patterns as Prognostic Tools for Canine Cutaneous Mast Cell Tumors. Vet. Pathol..

[19] Webster J.D., Yuzbasiyan-Gurkan V., Kaneene J.B., Miller R., Resau J.H., Kiupel M. (2006). The role of c-KIT in tumorigenesis: Evaluation in canine cutaneous mast cell tumors. Neoplasia.

[20] Garrett L.D. (2014). Canine mast cell tumors: Diagnosis, treatment, and prognosis. Vet. Med..

[21] Donnelly S., Davis M.P., Walsh D., Naughton M. (2002). World Health Organization Morphine in cancer pain management: A practical guide. Support Care Cancer.

[22] Berry S.H. (2015). Analgesia in the Perioperative Period. Vet. Clin. N. Am. Small Anim. Pract..

[23] Hermens J.M., Ebertz J.M., Hanifin J.M., Hirshman C.A. (1985). Comparison of histamine release in human skin mast cells induced by morphine, fentanyl, and oxymorphone. Anesthesiology.

[24] Nguyen J., Luk K., Vang D., Soto W., Vincent L., Robiner S., Saavedra R., Li Y., Gupta P., Gupta K. (2014). Morphine stimulates cancer progression and mast cell activation and impairs survival in transgenic mice with breast cancer. Br. J. Anaesth..

[25] Chéret J., Gherardini J., Soeberdt M., Hundt J.E., Abels C., Bertolini M., Paus R. (2020). Non-neuronal kappa-opioid receptor activation enhances epidermal keratinocyte proliferation, and modulates mast cell functions in human skin ex vivo. J. Dermatol..

[26] Yamamizu K., Furuta S., Hamada Y., Yamashita A., Kuzumaki N., Narita M., Doi K., Katayama S., Nagase H., Yamashita J.K. (2013). к Opioids inhibit tumor angiogenesis by suppressing VEGF signaling. Sci. Rep..

[27] Thompson J.J., Yager J.A., Best S.J., Pearl D.L., Coomber B.L., Torres R.N., Kiupel M., Foster R.A. (2011). Canine subcutaneous mast cell tumors: Cellular proliferation and KIT expression as prognostic indices. Vet. Pathol..

[28] Ploton D., Menager M., Jeannesson P., Himber G., Pigeon F., Adnet J.J. (1986). Improvement in the staining and in the visualization of the argyrophilic proteins of the nucleolar organizer region at the optical level. Histochem. J..

[29] Heel R.C., Brogden R.N., Speight T.M., Avery G.S. (1978). Butorphanol: A review of its pharmacological properties and therapeutic efficacy. Drugs.

[30] Trescot A.M., Datta S., Lee M., Hansen H. (2008). Opioid pharmacology. Pain Physician.

[31] Lennon F.E., Mirzapoiazova T., Mambetsariev B., Poroyko V.A., Salgia R., Moss J., Singleton P.A. (2014). The Mu opioid receptor promotes opioid and growth factor-induced proliferation, migration and Epithelial Mesenchymal Transition (EMT) in human lung cancer. PLoS ONE.

[32] Gupta K., Kshirsagar S., Chang L., Schwartz R., Law P.Y., Yee D., Hebbel R.P. (2002). Morphine stimulates angiogenesis by activating proangiogenic and survival-promoting signaling and promotes breast tumor growth. Cancer Res..

[33] Yaksh T.L., Eddinger K.A., Kokubu S., Wang Z., DiNardo A., Ramachandran R., Zhu Y., He Y., Weren F., Quang D. (2019). Mast Cell Degranulation and Fibroblast Activation in the Morphine-induced Spinal Mass: Role of Mas-related G Protein-coupled Receptor Signaling. Anesthesiology.

[34] Blunk J.A., Schmelz M., Zeck S., Skov P., Likar R., Koppert W. (2004). Opioid-induced mast cell activation and vascular responses is not mediated by mu-opioid receptors: An in vivo microdialysis study in human skin. Anesth. Analg..

[35] Kandefer-Gola M., Madej J.A., Dzimira S., Nowak M., Janus I., Ciaputa R. (2015). Comparative analysis of markers of cell proliferation in canine mast cell tumours according to current classifications. Pol. J. Vet. Sci..

[36] Meacham C.E., Morrison S.J. (2013). Tumour heterogeneity and cancer cell plasticity. Nature.

[37] Shaw T., Kudnig S.T., Firestone S.M. (2018). Diagnostic accuracy of pre-treatment biopsy for grading cutaneous mast cell tumours in dogs. Vet. Comp. Oncol..

[38] Curley T.L., Thamm D.H., Johnson S.W., Boscan P. (2021). Effects of morphine on histamine release from two cell lines of canine mast cell tumor and on plasma histamine concentrations in dogs with cutaneous mast cell tumor. Am. J. Vet. Res..

[39] The F.O., Buist M.R., Lei A., Bennink R.J., Hofland J., van den Wijngaard R.M., de Jonge W.J., Boeckxstaens G.E. (2009). The role of mast cell stabilization in treatment of postoperative ileus: A pilot study. Am. J. Gastroenterol..

[40] Blackwood L., Murphy S., Buracco P., De Vos J.P., De Fornel-Thibaud P., Hirschberger J., Kessler M., Pastor J., Ponce F., Savary-Bataille K. (2012). European consensus document on mast cell tumours in dogs and cats. Vet. Comp. Oncol..

